# Mutant huntingtin exon 1 protein detected in mouse brain with neoepitope antibody: effects of CAG repeat expansion, MutS Homolog 3 silencing and aggregation

**DOI:** 10.1093/braincomms/fcaf314

**Published:** 2025-08-29

**Authors:** Ellen Sapp, Adel Boudi, Andrew Iwanowicz, Jillian Belgrad, Rachael Miller, Riannon Robertson, Daniel O’Reilly, Ken Yamada, Yunping Deng, Marion Joni, Xueyi Li, Kimberly Kegel-Gleason, Anastasia Khvorova, Anton Reiner, Neil Aronin, Marian DiFiglia

**Affiliations:** Department of Neurology, Massachusetts General Hospital, Charlestown, MA 02129, USA; Department of Neurology, Massachusetts General Hospital, Charlestown, MA 02129, USA; Department of Neurology, Massachusetts General Hospital, Charlestown, MA 02129, USA; RNA Therapeutics Institute, University of Massachusetts Chan Medical School, Worcester, MA 01605, USA; Department of Medicine, University of Massachusetts Chan Medical School, Worcester, MA 01605, USA; Department of Neurology, Massachusetts General Hospital, Charlestown, MA 02129, USA; RNA Therapeutics Institute, University of Massachusetts Chan Medical School, Worcester, MA 01605, USA; RNA Therapeutics Institute, University of Massachusetts Chan Medical School, Worcester, MA 01605, USA; Department of Anatomy and Neurobiology, University of Tennessee Health Science Center, Memphis, TN 38163, USA; Department of Anatomy and Neurobiology, University of Tennessee Health Science Center, Memphis, TN 38163, USA; Department of Neurology, Massachusetts General Hospital, Charlestown, MA 02129, USA; Department of Neurology, Massachusetts General Hospital, Charlestown, MA 02129, USA; RNA Therapeutics Institute, University of Massachusetts Chan Medical School, Worcester, MA 01605, USA; Department of Anatomy and Neurobiology, University of Tennessee Health Science Center, Memphis, TN 38163, USA; Department of Medicine, University of Massachusetts Chan Medical School, Worcester, MA 01605, USA; Department of Neurology, Massachusetts General Hospital, Charlestown, MA 02129, USA

**Keywords:** HTT1a, western blot, MSH3 silencing, smear

## Abstract

*HTT1a* was identified in human and mouse Huntington’s disease brain as the pathogenic exon 1 mRNA generated from aberrant splicing between exon 1 and 2 of *HTT* that contributes to aggregate formation and neuronal dysfunction. Detection of the huntingtin exon 1 protein (HTT1a) has been accomplished with Meso Scale Discovery, Homogeneous Time Resolved Fluorescence and immunoprecipitation assays in Huntington’s disease knock-in mice, but direct detection in homogenates by gel electrophoresis and western blot assay has been lacking. Subcellular fractions prepared from mouse and human Huntington’s disease brain were separated by gel electrophoresis and probed by western blot with neoepitope monoclonal antibodies 1B12 and 11G2 directed to the C-terminal eight residues of HTT1a. In caudate putamen of an allelic series of 6-month-old Huntington’s disease knock-in mice (Q50, Q80, Q111, Q140 and Q175), HTT1a migration was inversely correlated with CAG repeat length and appeared as a sodium dodecyl sulphate soluble high molecular mass smear in Q111, Q140 and Q175 mice but weakly in Q80 and not in wild-type mice or Q50 indicating a CAG repeat size threshold for detecting HTT1a. HTT1a immunoreactivity diminished if 1B12 and 11G2 antibodies were preincubated with an eight amino acid peptide containing the C-terminus of HTT1a but not with an unrelated peptide sequence. Migration of HTT1a and its high molecular mass smear changed with age in caudate putamen of Q111, Q175 and YAC128 mice. Reducing levels of MutS Homolog 3 (MSH3) protein >84% in Q111 mice caudate putamen with small interfering RNA to *MSH3*, a modifier of CAG repeat expansion, significantly reduced levels of the high molecular mass smear suggesting that the effects of curbing CAG repeat expansion on HTT1a were quantifiable. A prominent 56–60 kDa doublet detected by 1B12 and 11G2 antibodies in lysates from human Huntington’s disease brain was not blocked by preincubation with C-terminal HTT1a blocking peptide and also appeared in brains of Parkinson’s disease patients. 1B12 and 11G2 antibodies did not immunoprecipitate huntingtin (HTT) proteins from either Huntington’s disease mouse or human brain lysates using conditions that pulled down full-length HTT with anti-HTT antibody 2B7. Altogether, these data show that 11G2 and 1B12 antibodies can be used in western blot assays to track and quantify immunoreactive HTT1a levels, solubility and subcellular localization in Huntington’s disease mouse brain.

## Teaser

Sapp *et al*. report that pathogenic exon 1 protein HTT1a is detected in brain of mouse models of Huntington’s disease by direct western blot assay using monoclonal antibodies 11G2 and 1B12. Lowering *MSH3* mRNA in the caudate putamen to prevent CAG repeat expansion reduced levels of HTT1a.

## Graphical Abstract

**Graphical Abstract:**
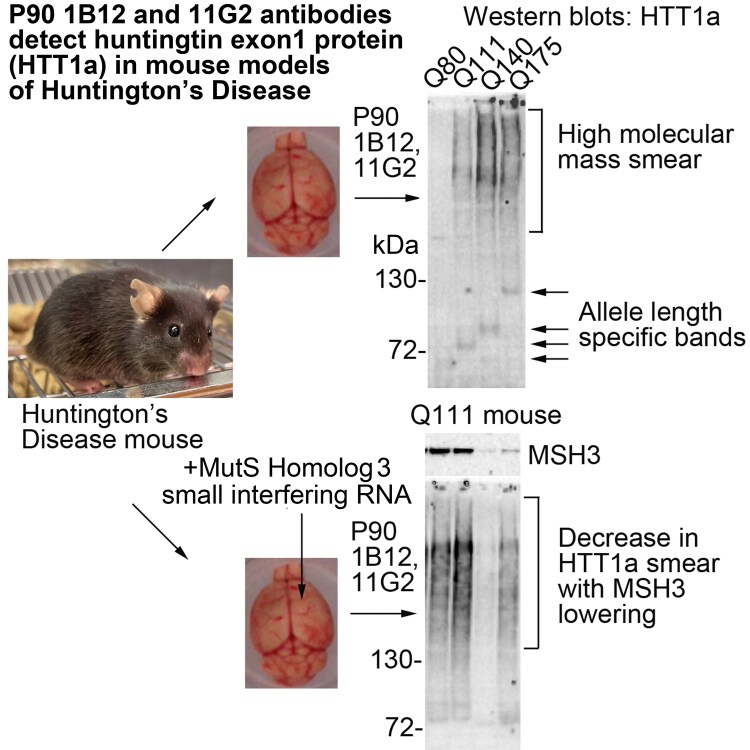

## Introduction

An mRNA arising from aberrant splicing between huntingtin exon 1 and 2 generates a polyadenylated mRNA named *HTT1a*.^1^  *HTT1a* transcript translated into the huntingtin exon 1 protein (HTT1a) in multiple Huntington’s disease mouse models including Q50, Q100 and Q150 mice, Q80 and zQ175 mice, as well as YAC128 mice and in the complete allelic series of Huntington’s disease knock-in mouse models.^1-8^  *HTT1a* is also detected in Huntington’s disease patient brain and fibroblasts.^9^  *HTT1a* mRNA was proposed to give rise to the toxic huntingtin exon 1 protein expressed in R6/2 transgenic mice which are more severely affected than Huntington’s disease knock-in mice expressing full-length huntingtin (HTT) because they die prematurely between 12 weeks and 6 months depending on the CAG repeat size.^10,11^ The MW8 antibody made to the C-terminal eight amino acids of exon 1, AEEPLHRP, has been used as a surrogate marker of HTT1a in immunoprecipitation assays, Meso Scale Discovery (MSD) and Homogeneous Time Resolved Fluorescence (HTRF) assays.^1,12^ When paired with antibody 4C9 or 2B7 in HTRF assays, antibody MW8 detects soluble and aggregated forms, respectively, of HTT1a.^3,13^ S830 and MW8 antibodies, which detect aggregates in brain of Huntington’s disease mice, were used to identify HTT1a in lysates immunoprecipitated from Q150 mouse brain with poly-glutamine antibody 3B5H10.^1^ MSD and HTRF assays can be costly and require considerable mouse brain tissue.^3^ A sensitive and direct detection of HTT1a by sodium dodecyl sulphate–polyacrylamide gel electrophoresis (SDS-PAGE) and western blot would provide a rapid, cost-effective method that utilizes smaller tissue samples.

Although HTT1a has been identified in Huntington’s disease knock-in mice and YAC128 transgenic mice using HTRF, MSD and immunoprecipitation assays,^3,9,14,15^ its direct detection by western blot has been elusive. Monoclonal neoepitope antibodies (P90 1B12 and 11G2) directed to the C-terminus 83–90 of HTT1a (based on HTT with 23 Qs) recently developed and characterized,^16-18^ that are highly sensitive for recognizing purified recombinant HTT1a by western blot compared to other antibodies directed to *N*-terminal HTT fragments, such as MW8, offered the possibility of detecting HTT1a using SDS-PAGE and western blot. Here, we tested the sensitivity of neoepitope antibodies to identify HTT1a by SDS-PAGE and western blot in brain samples from YAC128 transgenic mouse and Huntington’s disease knock-in mice from an allelic series (Q50, Q80, Q111, Q140 and Q175) and compared the results with R6/2 transgenic Huntington’s disease mice which express human exon 1 HTT. In Huntington’s disease knock-in mouse models, HTT1a appeared as a band and as a high molecular mass (HMM) smear measurable by densitometry. The migration and solubility of HTT1a in SDS-PAGE depended on CAG repeat length, subcellular compartment and age of mice. Silencing MutS Homolog 3 (*MSH3*) mRNA which encodes a mismatch repair protein that drives CAG expansion in Q111 mice attenuated HTT1a smear in caudate putamen, suggesting the use of 1B12 and 11G2 antibodies as a sensitive quantifiable readout for HTT1a aggregation.

## Materials and methods

### Sources of human and mouse brain tissue

Supplementary Table 1 lists the human brain tissue, regions, sources and references to our prior publications where brain tissues were used.^19-21^ All human tissues were de-identified and stored frozen at −80°C for at least 10 years and age, sex and CAG repeat length determined from PCR analysis of brain tissue are noted. Huntington’s disease Vonsattel grade of striatal pathology^22^ is included in the table when available. No change was seen in signal intensity of HTT when compared to the results in an earlier study where many of the same samples had been analysed by western blot.^19^

Mouse brain lysates from prior studies were prepared and stored frozen for one to 10 years.^23-26^ The time of storage at −80°C did not change HTT migration or signal intensity by western blot. The animal protocols for these studies were approved by the Massachusetts General Hospital (MGH) Subcommittee on Research Animal Care (SRAC)-Office of Laboratory Animal Welfare (OLAW) protocol #2004N000248 and University of Massachusetts Chan Medical School Institution Animal Care and Use Committee (IACUC PROTO202000010). All procedures conform to the United States Department of Agriculture Animal Welfare Act, the ‘Institute for Laboratory Animal Research Guide for the Care and Use of Laboratory Animals’, and followed institutional guidelines. Huntington’s disease knock-in mice (Q50, Q80, Q111, Q140 and Q175) were obtained from Jackson laboratories. These mice have the mouse exon 1 replaced with a mutant version of human exon 1 and are on C57BL/6 background. CAG repeat determined from tail DNA was provided by the supplier at the time of delivery and the range for each Huntington’s disease mouse line is reported in Table 1. YAC128 mice are on FVB background. For wild-type (WT), Q111, Q175 and YAC128 mice, male and female mice were used. For WT, Q50, Q80, Q111, Q140 and Q175 allelic series mice, only male mice were used. The experimenters were not blinded to the genotypes or treatment conditions of the mice. A total of 96 mice were used in this study and no samples were excluded. Mice were euthanized and the brain was removed and fresh-frozen or a cardiac perfusion with PBS was performed before brain removal and freezing at −80°C. The cortex from a 12-week-old R6/2 mouse on C57BL/6 background was provided by Dr Vanita Chopra.^27^ The CAG repeat number of 135 was determined at the MGH Genomics Core by PCR and capillary electrophoresis of PCR products.

**Table 1:** Changes in molecular mass of HTT1a protein in western blots probed with antibody 1B12: effects of CAG repeat number and gel type in Huntington’s disease mice with different CAG repeats

HD Mouse model	CAG repeats	Molecular mass of HTT1a protein	
		3–8% Tris-acetate gel	12% Bis-Tris gel
R6/2	135	85–90 kDa	60 kDa
Q80	84	65 kDa	
Q111	108–119	72–80 kDa	56 kDa
Q140	∼140	100 kDa	
Q175	185–202	130–140 kDa	90 kDa
YAC128	∼128	80 kDa	

For analysis of *MSH3* silencing, samples of an equal number of male and female Q111 mouse caudate putamen from a prior study^24^ and from a new cohort of mice that were injected bilaterally (125 µg per ventricle) in the lateral ventricle with di-valent siRNA directed to a non-targeting control (NTC) or *MSH3* (MSH3-1000) at 3 months and sacrificed at 5 months were used. Sequence and chemistry of the siRNAs were previously published.^24^ Mice with confirmed genotypes were randomly assigned to treatment groups, with males and females balanced. To help control for variability between injection sessions, the mice in each injection session were injected with either NTC or siRNA targeting *MSH3*. Mice were anaesthetized with isoflurane and monitored throughout the entire intracerebroventricular injection procedure. The mice were maintained on a heat pack the entire time under anaesthesia. When the mice were no longer responsive to a toe pinch, fur from the head was shaved, and the mice were placed on the stereotactic frame. A stereotactic procedure was used to locate the lateral ventricle. In brief, the skull was exposed using aseptic technique, bregma was identified, and the stereotactic coordinates were used to locate the skull region over the location of the lateral ventricle (mediolateral ±1 mm, posterior −0.2 mm, ventral −2.5 mm). A burr hole was drilled at the appropriate medial-lateral position, and the 33-gauge needle was positioned at ventral −2.5 mm. The divalent siRNA was infused at a rate of 750 nL/min for a total of 2.5 μL. This procedure was repeated in the other hemisphere. A total volume of 5 μL was delivered to each mouse. After the bilateral injections were completed, the skin incision was sutured, and the mice received a subcutaneous injection of meloxicam ER. The mice were placed on a heat pack in a recovery cage, monitored until sternal and then checked daily for the first 72 h post-procedure and weekly throughout the study. After brains were harvested, they were cut into 1 mm coronal blocks. The integrity of the ventricular spaces and the brain surrounding the lateral ventricles at the level of the caudate putamen was checked. The trajectory of the needle track could be followed in some brains. When routine histology was performed, the lateral ventricle and adjacent tissue were inspected to verify its integrity. Needle punches of the caudate putamen were taken from brain slices to make crude homogenates (CHs, see below).

### Preparation of lysates for sodium dodecyl sulphate–polyacrylamide gel electrophoresis from human and mouse brains.

Lysates from brain tissue were prepared using one of two basic methods reported in our prior studies.^25,28,29^ Pieces of human putamen or cortex (1–2 mm^3^) or mouse caudate putamen were homogenized in 100–200 μL 10 mM N-2-hydroxyethylpiperazine-N′-2-ethanesulfonic acid (HEPES) pH 7.2, 250 mM sucrose, 1 mM ethylenediaminetetraacetic acid (EDTA) + protease inhibitor tablet (Roche) + 1 mM NaF + 1 mM Na_3_VO_4_ in an Eppendorf tube with a plastic pestle. An aliquot of the CH was removed and sonicated for 10 s. In some cases, the sample was centrifuged at 800xg for 15 min at 4°C. The supernatant (S1) from this spin corresponds to the cytoplasmic fraction, and the pellet (P1), which was resuspended in the same buffer, includes nuclear proteins. For most experiments reported here, the CH was used as indicated in figure legends.

A second method of preparation of brain lysates required a different buffer. In brief, the entire caudate putamen per mouse brain was homogenized in 3 mL of 0.32M sucrose, 10 mM dithiothreitol (DTT) + protease inhibitor tablet (Roche) and 100 µL was removed as a CH. Some lysates that had been prepared in this manner were part of a published study of 6-month-old WT, Q50, Q80, Q111, Q140 and Q175 mice.^25^ The remaining samples that were stored frozen were used in the current study.

Protein extraction using mild detergent was tested by homogenizing 6-month-old WT and Q111 caudate putamen on ice in 20 mM Tris pH 7.4, 150 mM NaCl, 1% Triton X-100 and 0.1% NP40, incubating 15 min on ice then centrifuging at 16 000xg 15 min as 4°C following our previous method.^30^ The supernatant was removed and the pellet was resuspended in the same buffer. Twenty micrograms was separated by SDS-PAGE for western blot analysis.

To solubilize aggregated HTT, two methods were tested including our previously published protocol.^21^ Specifically, 20 µg of CH prepared in 10 mM HEPES pH 7.2, 250 mM sucrose and 1 mM EDTA as described above from cortex of R6/2 mice and caudate putamen of 6-month-old WT and Q175 mice were incubated in 100 mM DTT and 8 M urea for 30 min at room temperature with vortexing every 5 min before adding lithium dodecyl sulphate (LDS) sample buffer (Invitrogen) and separating by SDS-PAGE. A second method described by Landles *et al*.^15^ uses a sequential treatment with SDS followed by formic acid. Fifty micrograms of R6/2 cortex CH was centrifuged at 13 000xg for 15 min at 4°C, and the pellet was resuspended in 50 µL 1 × SDS buffer (2% SDS, 5% beta-mercaptoethanol, 15% glycerol). Samples were boiled for 10 min, sonicated for 20 s and centrifuged at 13 000xg for 15 min at 4°C, and the supernatant was removed as the SDS soluble fraction. One hundred microlitres formic acid was added to the pellets, and the samples were incubated at 37C with shaking at 250 rpm for 1 h, then dried overnight under vacuum in a desiccator. Dried formic acid pellets were neutralized with 1 M Tris pH 8.6 before adding LDS sample buffer and boiled for 10 min, and the entire sample was separated by SDS-PAGE as described below.

### Sodium dodecyl sulphate–polyacrylamide gel electrophoresis and western blot

Protein concentration was determined using the Bradford assay (Bio-Rad Protein assay). Equal amounts of protein as indicated in the figure legend (typically 20 μg) were prepared in 1 × LDS buffer (Invitrogen) + 100 mM DTT, boiled for 5 min and separated by SDS-PAGE using 3–8% Tris-acetate (NuPAGE 15-well, 1.5 mm, Invitrogen or Criterion 26-well, 1.0 mm, Bio-Rad) with Tricine running buffer (Bio-Rad) or 4–12% Bis-Tris gels (Criterion 26-well, 1.0 mm, Bio-Rad) or 12% Bis-Tris gels (NuPAGE 15-well, 1.0 mm, Invitrogen or Criterion 26-well, 1.0 mm, Bio-Rad) with MOPS running buffer (Bio-Rad), at 120 V until the dye-front reached the bottom of the gel (∼1.5 h); then gels were soaked in Tris-glycine transfer buffer + 0.1% SDS for 5 min before transfer. We compared wet transfers at 100 V for 1 h and 35 V overnight to our normal transfer using TransBlot Turbo (Bio-Rad) and did not see an appreciable change in the detection of the HMM protein, so we transferred proteins to nitrocellulose with the TransBlot Turbo apparatus at high molecular weight setting (25 V for 10 min) throughout this study. Blots were incubated in blocking buffer [5% blotting-grade blocker (Bio-Rad) in Tris-buffered saline + 0.1% Tween-20 (TBST)] for 1 h at room temperature, then in primary antibody diluted in blocking buffer at 4°C overnight with agitation. For controls using blocking and unrelated peptides, antibodies were diluted to 5 µg/mL in blocking buffer and incubated with agitation with the HTT1a peptide or unrelated peptide at 26 µM for 1 h at room temperature for the human brain or overnight at 4°C for the mouse brain before applying to the nitrocellulose blot.

### Immunoprecipitation assays

Immunoprecipitation assays were performed based on our previously published protocol.^31^ Frozen mouse caudate putamen or pieces of human cortex were homogenized in GAL4/IP buffer (50 mM Tris pH 7.2, 250 mM NaCl, 5 mM EDTA, 1%NP40 plus protease inhibitor tablet (Roche), 1 mM NaF, 1 mM Na_3_VO_4_) and incubated on ice for 15 min before centrifugation at 13 000xg for 2 min at 4°C. The protein concentration of the supernatant was determined using the Bradford assay (Bio-Rad) and 5 tubes of 1 mg protein in 1 mL GAL4/IP buffer were prepared. Protein A/G Sepharose beads (Santa Cruz Biotechnology) were washed and blocked for 4 h or overnight in PBS + 1% bovine serum albumin. Brain lysates were precleared for 1 h in blocked Protein A/G beads before adding 5 µg 1B12, 11G2 or anti-HTT antibody 2B7 or normal rabbit or mouse IgG and incubating overnight at 4°C. To each tube, 30 µL pre-blocked Protein A/G Sepharose diluted 1:1 in GAL4/IP buffer was added and incubated for 2 h with agitation at 4°C. Sepharose beads were then centrifuged at 4000 rpm for 4 min and washed 4 times in 750 µL GAL4/IP buffer. After final wash, Sepharose beads were resuspended in 30 µL 2 × LDS sample buffer + DTT, boiled for 5 min then 25 µL was analysed by western blot with 1B12 or anti-HTT antibodies.

### Sources of antibodies and peptides used for western blot

Antibodies and dilutions were as follows: anti-HTT antibody Ab1 (aa1-17,^32^ 1:2000, rabbit), MW8 (Developmental Studies Hybridoma Bank, University of Iowa, 1:500, mouse), S830 (generous gift from Dr Gillian Bates, 1:6000, sheep), 2B7 antibody (N-terminal HTT, Coriell Institute for Medical Research, 1:750, mouse), anti-HTT neo P90 antibodies (neoepitope to aa83-90, clones 1B12 and 11G2, HD Community BioRepository Collection, Coriell Institute for Medical Research, 5 µg/mL, rabbit), MSH3 (Santa Cruz BioTech #sc-271079, 1:500, mouse), glyceraldehyde-3-phosphate dehydrogenase (GAPDH, MilliporeSigma #MAB374, 1:10000, mouse) and B-actin (MilliporeSigma #A5441, 1:5000, mouse). Blots were washed in TBST, then incubated in peroxidase-labeled secondary antibodies diluted 1:2500 for rabbit IgG and 1:5000 for mouse and sheep IgG in blocking buffer for 1 h at room temperature. Immunoprecipitation blots were incubated in rabbit or mouse IgG, light-chain specific (Cell Signaling), diluted 1:2000 in blocking buffer. Blots were washed in TBST, and bands were visualized using SuperSignal West Pico PLUS Chemiluminescent substrate (Thermo Scientific) and ChemiDoc XRS + with Image Lab software (Bio-Rad). Some blots were stripped for 30 min with Restore stripping buffer (Thermo Scientific) before washing, blocking and re-probing with a different antibody. Peptides were obtained from Thermo Fisher. The sequences were AEEPLHRP for HTT1a C-terminus and YHRLLTCLRNVHKVTTC for the unrelated peptide.

### Pixel intensity quantification

Image analysis was performed using ImageJ software. For HTT, MSH3 and loading controls, bands were manually circled, and the area and average intensity were determined. The total signal intensity of each band was calculated by multiplying the area by the average intensity. For HTT1a levels in YAC128, allelic series and Q111 mice treated with *MSH3* siRNA, the average intensity of the smear and band was measured in equal areas for each lane. The dashed lines in one lane of western blots represent the areas measured in all lanes. For the allelic series, the average signal intensity of the smear obtained in the WT lanes was subtracted as background from the average signal intensity of the smear in the Q80, Q111, Q140 and Q175 samples. The signal intensities were normalized to the loading control signal of B-actin or GAPDH.

### Neuronal differentiation

Experiments were performed with oversight of Human Embryonic Stem Cell Research Oversight Committee (ESCRO Committee) through the MassGeneral Brigham Institutional Biosafety Committee (PIBC) (ESCRO#: 2015-01-02 and PIBC Reg# 2017B000023) using human induced pluripotent stem cells (iPSCs) CS09iCTR-109n4 (CS vial ID: 1034860) and CS09iCTR-109n5 (CS vial ID: 1034589) hereafter referred to as HD109Q (clone n4 and n5) described by Mattis *et al*., 2015.^33^ iPSCs were differentiated into cerebral cortex neurons as described^34,35^ with modifications. Briefly, neural induction occurred on confluent iPSCs plated on Matrigel (Corning, 354277) for 10 days in Neural Induction Medium. Neuroepithelial sheets were lifted with dispase (Stemcell Technologies, catalogue 07923) and plated on Matrigel. Fibroblast growth factor (FGF) treatment (20 ng/mL, Gibco, PHG0023) in Neural Maintenance Medium (NMM) was initiated on Day 14 and stopped at Day 18 where neural rosettes were observed. Upon successful neural development, cells were cultured continuously in NMM with brain derived neurotrophic factor (BDNF, 10 ng/mL, Gibco, PHC7074) and glial cell line derived neurotrophic factor (GDNF, 10 ng/mL, Gibco, PHC7075) 42 days after the start of the neural induction along with anti-mitotic inhibitors 5-fluoro-2′-deoxyuridine (1 µM, Sigma-Aldrich, F0503) and Uridine (1 µM, Sigma-Aldrich, U3750). Neuron cultures were characterized using an antibody panel by western blot and immunocytochemistry to endorse the proper neuronal differentiation. Neurons were harvested in 10 mM HEPES pH 7.2, 250 mM sucrose and 1 mM EDTA + protease inhibitor tablet (Roche) + 1 mM NaF + 1 mM Na_3_VO_4_ and homogenized on ice before determining protein concentration using the Bradford method (Bio-Rad).

### Statistical analysis

Statistical analyses were performed using GraphPad Prism v10.4.1 software. Data were tested for Gaussian distribution using the Shapiro–Wilk test. Those data sets with normal distribution were subject to unpaired *t*-tests or one-way ANOVA with Tukey’s multiple comparison test and the data set that was not normally distributed was subject to a Mann–Whitney test. Pearson *r* test was used for correlation analyses. Statistical test, sample size and *P*-values are listed in the figure legends.

## Results

### Detection and solubility of HTT1a protein in brain of Huntington’s disease knock-in mice and YAC128 transgenic mice are dependent on age and CAG repeat length

**Figure 1:**
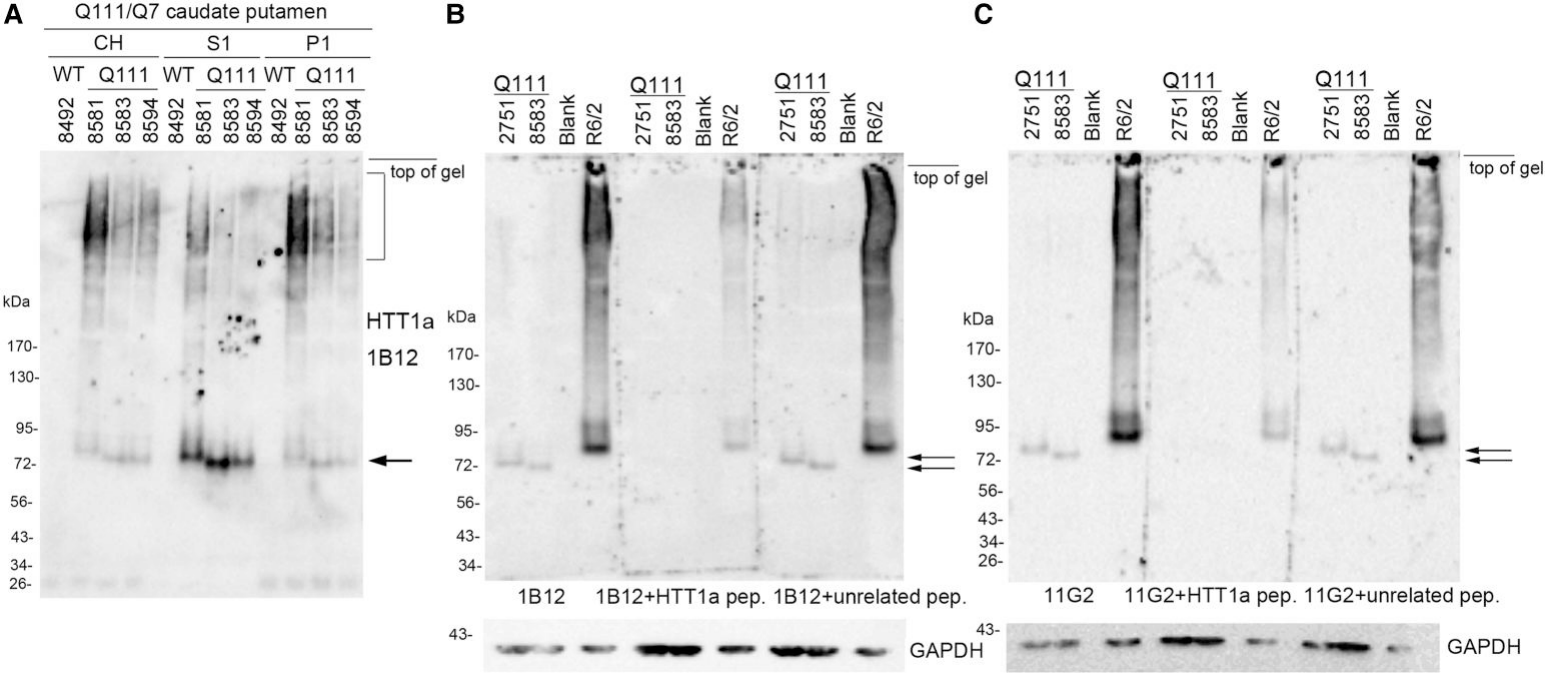
**Subcellular compartments and peptide blocking of HTT1a antibodies 1B12 and 11G2.** (**A**) CH as well as S1 and P1 fractions (20 μg each) from 6-month-old WT and Q111 mice were separated on a 3–8% Tris-acetate 26-well gel and probed with P90 antibody 1B12. HTT1a migrates to 75 kDa (arrow) and is most expressed in the S1 fraction whereas it appears as a HMM smear (bracket) in CH and P1 fractions. Note the absence of signal in WT mice. (**B** and **C**) 20 µg of 6-month-old Q111 caudate putamen S1 fraction samples 2751 (118 CAG repeats) and 8583 (113 CAG repeats) and 12-week-old R6/2 cortex CH were separated on 3–8% Tris-acetate 15 well gels. The signal at 72–75 kDa (at level of arrows) in Huntington’s disease Q111 mice is absent when antibodies 1B12 (**B**, middle blot) and 11G2 (**C**, middle blot) are preincubated with the HTT1a C-terminal peptide AEEPLHRP before western blot analysis. The intensity of the HTT1a bands and smear in the R6/2 cortex is greatly reduced by antibody preincubation with the blocking peptide. HTT1a detection by 1B12 (**B**) and 11G2 (**C**) antibodies is unaltered by preincubation with an unrelated peptide (compare left and right blots in **B** and **C**). At the top of the blots, mouse numbers are positioned at the centre of each lane. ‘Blank’ marks a lane without protein.

We examined subcellular compartments from Q111 Huntington’s disease mice which belong to an allelic series that has mouse exon 1 replaced with a mutant version of human exon 1 and one 12-week-old R6/2 mouse that has a human exon 1 transgene (Fig. 1). The presence of HTT1a in CHs, S1 and P1 fractions of caudate putamen from 6-month-old WT and Q111 mice was analysed together by western blot using 3–8% Tris-acetate gradient gels for protein separation (Fig. 1A). HTT1a migrated as a discrete band to about 75 kDa in S1 fractions and as a HMM smear in CHs and P1 fractions (Fig. 1A). No signal was detected in WT mice. 1B12 and 11G2 antibodies detected immunoreactive HTT1a by western blot at around 72 kDa in soluble (S1) fractions prepared from caudate putamen of two Q111 Huntington’s disease knock-in mice and around 80 kDa in one R6/2 mouse cortex. These bands were absent in Q111 samples or significantly diminished in R6/2 lysates by preincubation with a C-terminal HTT1a peptide (Fig. 1B and C). A broad HMM smear was also detected in the R6/2 lysates by 1B12 and 11G2 antibodies and markedly reduced by antibody preincubation with C-terminal HTT1a peptide. Preincubation with an unrelated peptide had no effect (Fig. 1B and C). HTT1a bands migrated faster if 12% Bis-Tris gels were used for protein separation (see Table 1 for molecular mass comparisons), and the smear in R6/2 was more compressed at the top of the blot (Supplementary Fig. 1). These results suggest the immunoreactive bands and smear detected by 1B12 and 11G2 recognized HTT1a. To further support specificity, immunoprecipitation assays were performed. Antibodies 11G2 and 1B12 did not immunoprecipitate HTT1a in brain lysates from Huntington’s disease Q140 or Q111 mice whereas under the same conditions full-length WT and mutant HTT were immunoprecipitated by antibody 2B7 (Supplementary Fig. 2). To determine if HTT1a detection in brains of Huntington’s disease knock-in mice could be improved by methods that solubilize aggregates,^15^ lysates were treated with urea, or SDS and formic acid, or by using a gentler extraction in Tris buffer with Triton X-100 and NP40 (Supplementary Fig. 3A-D). Urea and gentle extraction preparations of the lysates did not improve detection of the HTT1a band. However, solubilization with SDS alone increased the intensity of the HMM HTT1a smear and a HMM band (Supplementary Fig. 3D, small arrow) but not the 85 kDa HTT1a band (large arrow). After addition of formic acid following SDS treatment, HTT1a immunoreactivity appeared as a conical shaped smear that migrated to the level of HTT1a without a discrete band. It is possible that the overnight drying time we used compared to the 4 h used by Landles *et al*.^15^ affected HTT1a migration and/or caused some HTT1a degradation.

**Figure 2:**
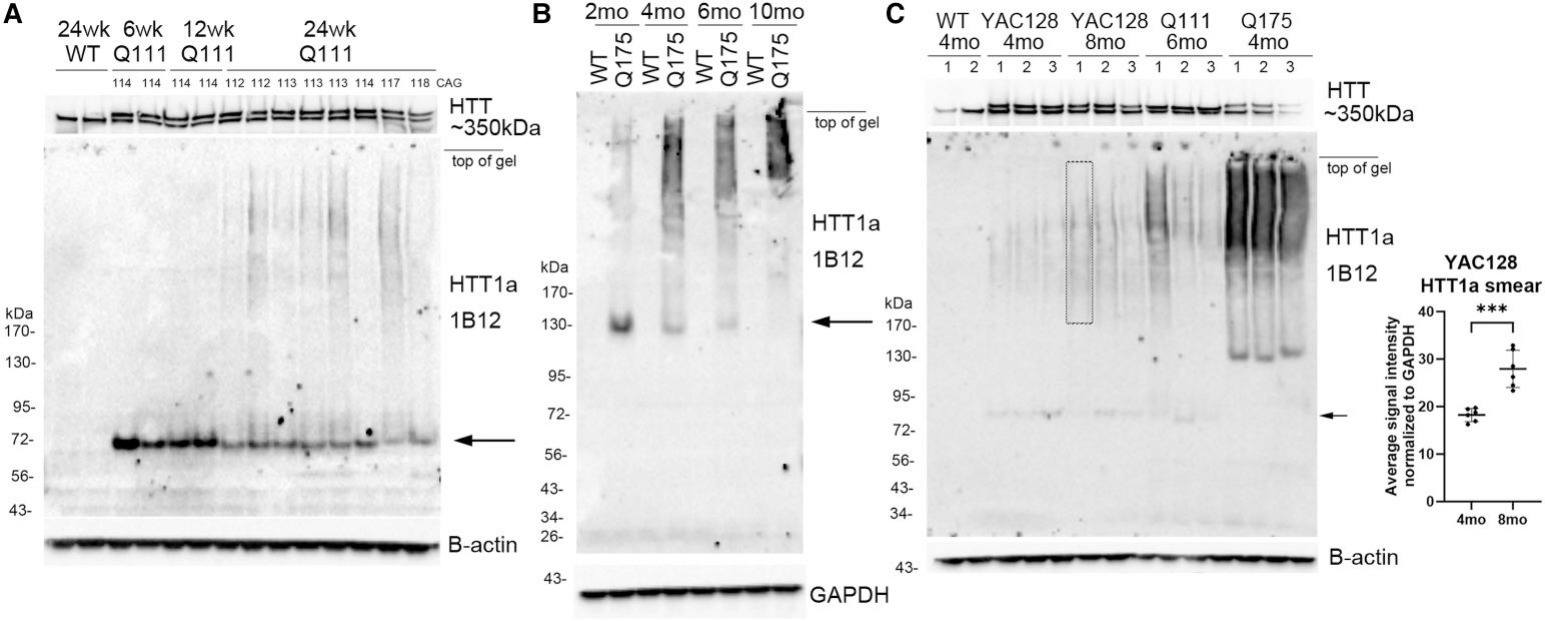
**Effects of age on HTT1a expression in Q111, Q175 and YAC128 mice.** Changes in levels, molecular mass and SDS solubility. (**A**) Western blot of S1 fractions of caudate putamen from WT mouse at 24 weeks and Q111 mice at 6, 12 and 24 weeks. 20 µg samples were separated on a 15-well 3–8% Tris-acetate gel. HTT1a migrates to about 75 kDa (arrow), and intensity decreases progressively between 6 weeks and 24 weeks. Note that at 24 weeks HTT1a smear appears in Q111 mice but not in WT mice. CAG repeat was determined from tail DNA and is indicated at the top of the blot. Blot was re-probed with anti-HTT antibody Ab1 to detect full-length HTT. (**B**) Western blot of CHs of WT and Q175 mice at 2, 4, 6 and 10 months. 20 µg samples were separated on a 3–8% Tris-acetate 26-well gel. HTT1a band migrates to about 130 kDa (arrow), and intensity decreases with increased age from 2 months to 10 months. In contrast the presence of HMM HTT1a smear increases with age. HTT1a is not detected in the WT mice. (**C**) Western blot of CHs prepared from caudate putamen of 4- and 8-month-old YAC128 mice show a significant increase in the HTT1a smear from 4 to 8 months (***P = 0.0002, *t* = 5.692, df = 10, two-tailed unpaired *t*-test, *n* = 6 per group; each dot represents one mouse and bars are mean ± SD). Three of the six samples are shown in this figure and box in 8mo #1 shows area measured in all mice. Images for analysis of *n* = 6 samples per group are shown in Supplementary Fig. 3E. The HTT1a band in the YAC128 mice runs at about 80 kDa (small arrow) which is slightly larger than in the Q111 mice, as expected. Note that the loading controls, which are B-actin for **A** and **C** and GAPDH for **B**, show equal signal intensities indicating equal protein loading. At the top of the blots, mouse numbers or CAG repeats are positioned at the centre of each lane. See Supplementary Fig. 5 for uncropped blots.

Next, the effect of age on HTT1a expression was explored in different Huntington’s disease mouse models. In S1 fractions from 6-, 12- and 24-week-old Q111 mice compared in the same western blot, HTT1a migrated to about 75 kDa (Fig. 2A, arrow), but levels diminished with age to about 50% at 24 weeks when HTT1a solubility was replaced by a HMM smear (Fig. 2A). No smear appeared in WT mice lysates at 24 weeks. HTT1a was also examined in Q175 mice of different ages where it migrated to about 130 kDa (Fig. 2B, arrow) and decreased in intensity with age between 2 months and 10 months coinciding with a marked rise in the SDS soluble smear (Fig. 2B). HTT1a smear was also significantly increased at 8 months compared to 4 months in lysates from YAC128 mice (Fig. 2C; Supplementary Fig. 3E). Altogether these data showed that HTT1a band intensity by western blot was age-dependent and its migration slowed by SDS-PAGE with increased CAG repeat expansion, although other factors affecting HTT1a migration cannot be ruled out. Importantly, with increasing age, the pattern of HTT1a migration changed from a CAG repeat length-dependent band to a HMM smear.

**Figure 3:**
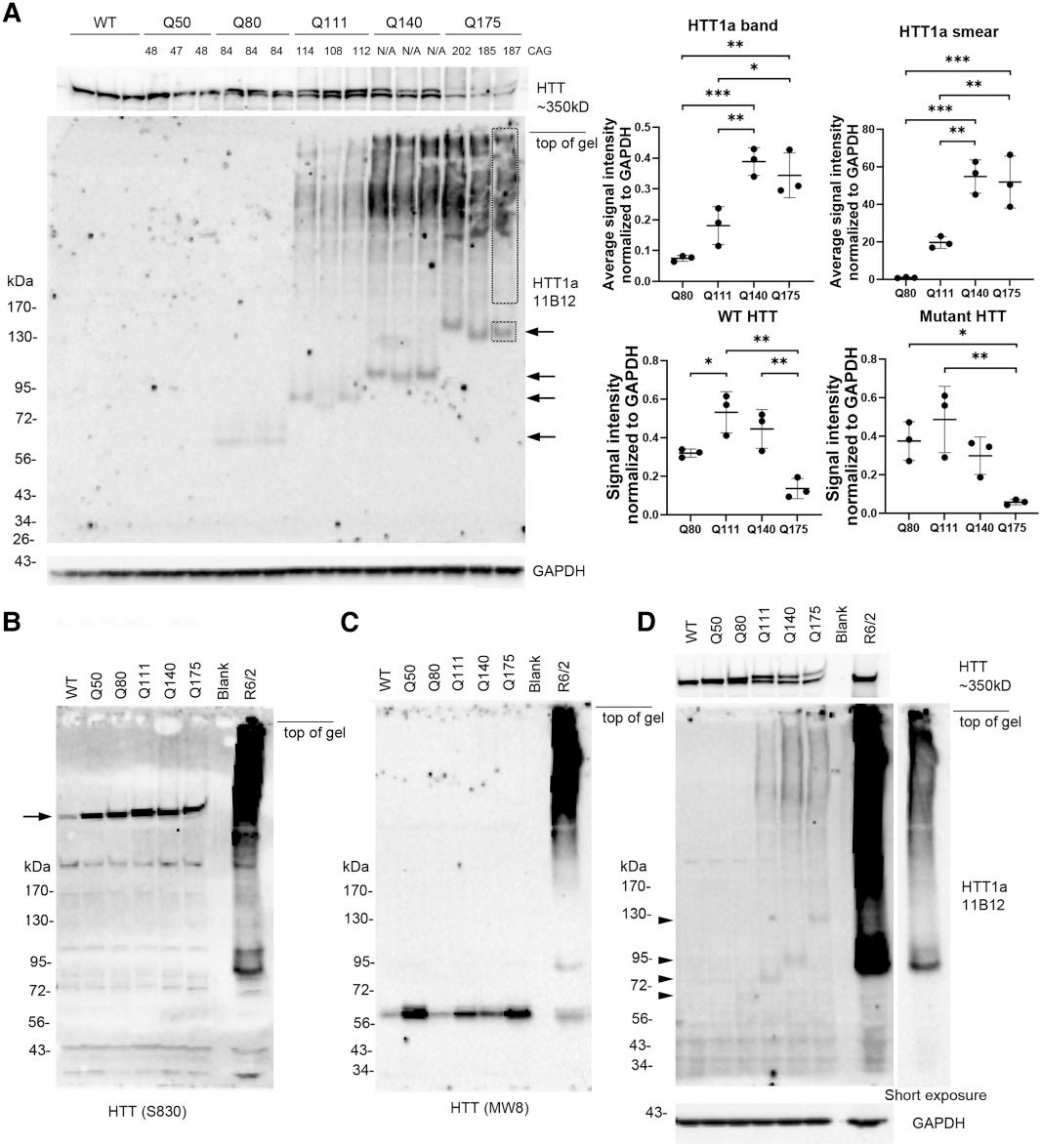
**Intensity, size and solubility of HTT1a in caudate putamen of 6-month-old Huntington’s disease knock-in mice allelic series.** (**A**) SDS-PAGE and western blot probed with antibody 1B12 in CHs from caudate putamen of WT, Q50, Q80, Q111, Q140 and Q175 mice. 15 µg samples were separated on 3–8% Tris-acetate 26-well gels. Numbers at the top are CAG repeats determined from tail DNA. Large blot shows HTT1a probed with 1B12 and the strip below is loading control for GAPDH. Migration of HTT1a (arrows) slows with increasing CAG repeat from about 65 kDa in Q80 to about 130 kDa in Q175 mice. HTT1a is not visible in WT or Q50 mice. HTT1a smear is seen in lysates from Q111, Q140 and Q175 mice. Strip above large blot is a re-probe with anti-HTT antibody Ab1 and shows WT and mutant HTT. Note decline in levels of full-length WT and mutant HTT in Q175 samples. Graphs at top right show average intensity of HTT1a and HTT1a smear normalized to GAPDH. Boxes in right lane of blot indicate the same areas that were measured in all lanes. The signal measured in WT lanes was subtracted out as background, and the signal for non-specific dots in the third lane of Q140 mice and second and third lane of Q175 mice was also subtracted. Bottom graphs show levels of WT and mutant HTT normalized to GAPDH (one-way ANOVA with Tukey’s multiple comparison test, HTT1a band: *F* = 22.75, *P* = 0.0003; HTT1a smear: *F* = 29.04, *P* = 0.00001; WT HTT: *F* = 14.30, *P* = 0.0014; mutant HTT: *F* = 89.029, *P* = 0.0085; *P < 0.05, **P < 0.01, ***P < 0.01, *n* = 3 per group; each dot represents one mouse and bars are mean ± SD). (**B–D**) Comparison of protein detection by antibodies S830, MW8 and 1B12. 15 µg CHs from *n* = 1 each 6-month WT, Q50, Q80, Q111, Q140, Q175 striatum and 12 week R6/2 cortex were separated on two 3–8% Tris-acetate 15-well gels, one probed with anti-HTT antibody S830 (**B**) and the other with anti-HTT antibody MW8 (**C**). The blot in **C** was stripped and re-probed with 1B12 antibody (**D**). In **B**, **C** and **D**, all three antibodies detect 1–2 bands in R6/2 mouse cortex at ∼90 and 100 kDa and a prominent HMM SDS soluble smear. S830 detects full-length HTT (arrow on left) in all cortex samples with a stronger signal for mutant than WT HTT. In **C**, MW8 detects an unknown band at ∼60 kDa in WT and Huntington’s disease knock-in mouse models. In **D**, 1B12 antibody detects HTT1a which migrates in relation to its CAG repeat: at 65 kDa for Q80, 75 kDa in Q111, 100 kDa in Q140 and 130 kDa in Q175 (arrowheads on left). No CAG length dependent bands are detected in Huntington’s disease knock-in mice with S830 or MW8 (**B** and **C**). At the top of the blots, mouse labels or CAG repeats are positioned at the centre of each lane. ‘Blank’ marks a lane without protein. See Supplementary Fig. 6 for uncropped blots.

We also compared HTT1a expression in Huntington’s disease knock-in mice that had different CAG repeats in exon 1 (Fig. 3A). CH from caudate putamen of 6-month-old WT, Q50, Q80, Q111, Q140 and Q175 mice showed differences in HTT1a migration when proteins were separated in 3–8% Tris-acetate gradient gels. HTT1a was ∼65 kDa in Q80 mice, 75 kDa in Q111 mice, 100 kDa in Q140 mice and 130 kDa in Q175 mice (Fig. 3A, small arrows, and Table 1). The intensity of HTT1a also significantly increased with CAG repeat size (graphs in Fig. 3A). A HTT1a HMM smear appeared in Q111, Q140 and Q175 mice and increased in signal intensity with increase in CAG repeat number (graphs in Fig. 3A). HTT1a was not detected in WT or Q50 mice. N-terminal HTT1-17 antibody Ab1 detected full-length WT and mutant HTT at about 350 kDa in all mice. In Q175 mice, full-length HTT levels were significantly lower suggesting that the marked HTT1a accumulation seen as a smear on western blot in these mice may affect levels of full-length HTT (Fig. 3A, top).

We wondered if the smear seen with 1B12 and 11G2 (in Fig. 1C) was also detected by other anti-HTT antibodies. S830 is a polyclonal sheep antibody raised against exon 1 with 53Q and MW8 is a monoclonal antibody made against exon 1 with 67Q. Both antibodies label aggregates by immunohistochemistry and MW8 has been used in HTRF based aggregation assays.^13,36^ All three antibodies detected a HMM smear in the R6/2 transgenic mice but only 1B12 detected a smear in samples from Huntington’s disease knock-in mice (Fig. 3B–D). This suggests 1B12 antibody is more sensitive than S830 and MW8 or may detect a unique conformation in HTT.

### The HTT1a smear decreases in caudate putamen of Q111 mice after lowering levels of MSH3, a mismatch repair protein

**Figure 4:**
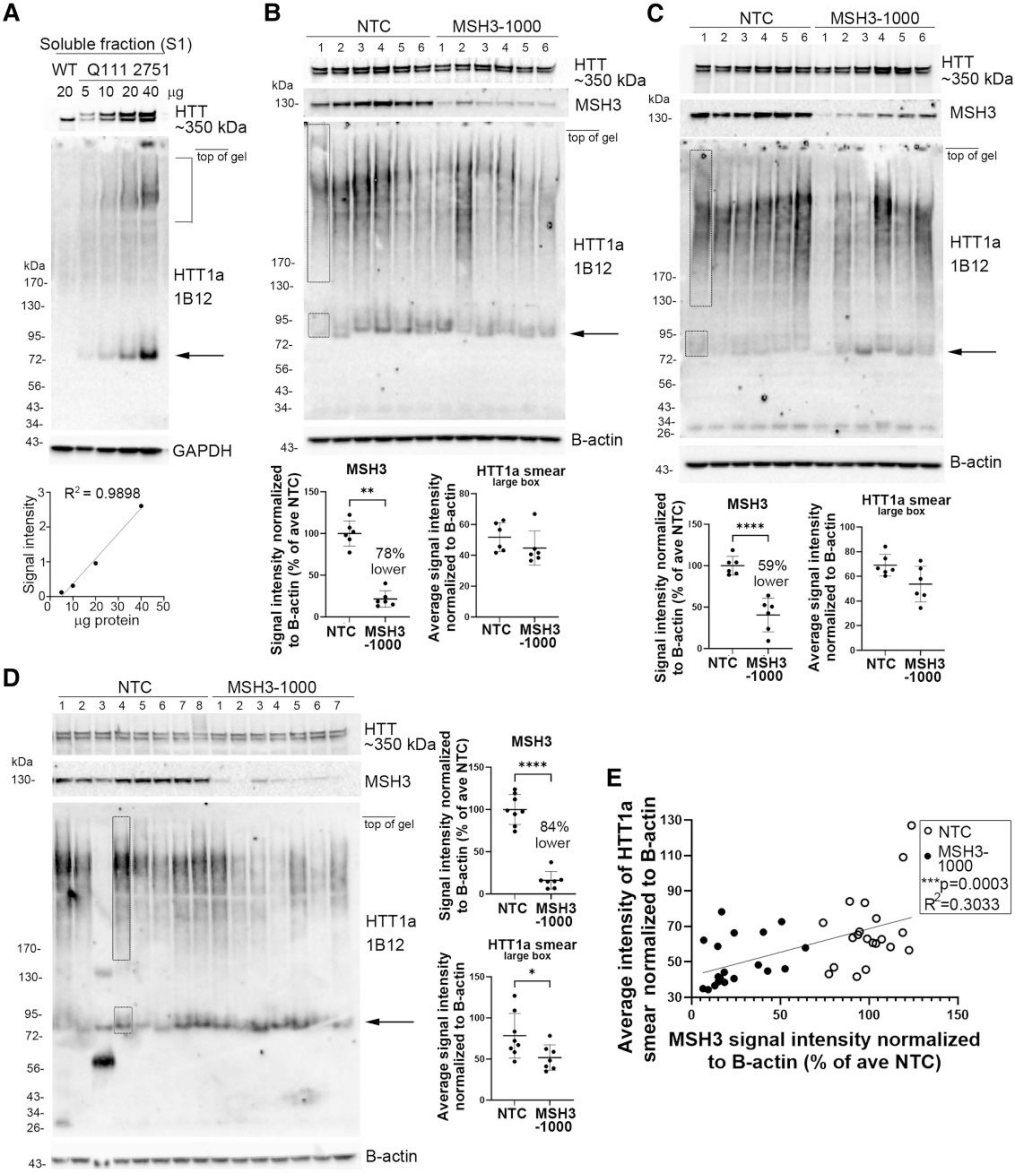
**Levels of HTT1a in Huntington’s disease mice caudate putamen: effects of protein concentration and treatment with siRNA to *MSH3*.** (**A**) Signal for HTT1a detected with 1B12 antibody is concentration dependent. S1 fraction from 6-month-old Q111 mouse 2751 was loaded at different protein concentrations for SDS-PAGE (5–40 µg) using a 3–8% Tris-acetate 15-well gel. Western blot probed with 1B12 shows signal for 75 kDa band (arrow) increases with increasing protein concentration [graph below, Pearson’s *r* test, *r* = 0.9949, *R*^2^ = 0.9898, *P* = 0.0051 (two-tailed)]. HTT1a smear at bracket is also concentration dependent. Similarly, full-length HTT detected with Ab1 (above) and GAPDH (below) increase with protein concentration. The 75 kDa band at level of arrow was detected in Q111 samples but not WT. (**B–D**) Effects of reducing levels of *MSH3* mRNA on HTT1a levels in CHs from 5-month-old Q111 mice. Blots from the same samples were probed with anti-HTT1a 1B12, anti-HTT Ab1 (aa1-17), anti-MSH3 and anti-B-actin antibodies. Three experiments show that HTT1a smear is reduced in most lanes where MSH3 levels are markedly reduced. Graphs show the level of MSH3 lowering with MSH3-1000 siRNA and average intensity of HTT1a smear where each dot represents one mouse and bars are mean ± SD. Equal areas of the smear and 75 kDa band (arrow) as indicated by dashed lines in the first lane in **B** and **C** and fourth lane in **D** were measured by densitometry. Graphs show that the HTT1a smear is significantly reduced in lysates from MSH3-1000 siRNA-treated mice compared to a NTC when the level of MSH3 lowering was >80% (**D**, *P = 0.0395, *t* = 2.289, df = 13, two-tailed unpaired *t*-test, *N* = 8 NTC and 7 MSH3). The signal intensity of 75 kDa band is not changed. (**E**) There is a significant correlation between MSH3 and HTT1a signal intensities when the data from the three experiments were combined [Pearson’s *r* test, *r* = 0.5507, *R*^2^ = 0.3033; ***P = 0.0003 (two-tailed), *n* = 20 NTC and 19 MSH3 mice; each dot represents one mouse]. 20 µg CH samples were separated on 3–8% Tris-acetate 15-well gels in **B** and **C** and a 26-well gel in **D**. At the top of the blots, mouse numbers are positioned at the centre of each lane. See Supplementary Fig. 7 for uncropped blots.

Results above in Huntington’s disease knock-in mice showed that detection of HTT1a by western blot was dependent on its subcellular compartment, age of mice and CAG repeat length. To determine if HTT1a detected with 1B12 antibody could be quantified by western blot assay, we compared different protein concentrations (5, 10, 20 and 40 µg) from the same Q111 mouse brain and performed densitometry. Results showed a concentration-dependent increase in signal for HTT1a, signifying the antibody was sufficiently sensitive to use for a quantitative assay (Fig. 4A).

The mismatch repair protein MSH3 is required for CAG repeat expansion and for nuclear localization of mutant HTT in Q111 mice^37^ but not in Q175 mice.^38^ Previously, we showed that treating 3-month-old Q111 mice with MSH3-1000, a divalent siRNA targeting *MSH3*, lowered MSH3 protein by 54% and inhibited CAG repeat expansion at 5 months compared to treatment with a NTC.^24^ In lysates from caudate putamen of three cohorts of mice treated by intraventricular injection with MSH3-1000 siRNA (N = 6–8 mice/group/study), there was a reduction in mean levels of MSH3 protein by 78% (Fig. 4B), 59% (Fig. 4C) and 84% (Fig. 4D). Western blot showed that the HTT1a smear was visually reduced in most of the Q111 Huntington’s disease mice treated with MSH3-1000 compared to NTC. However, a mean lowering of 84% MSH3 protein levels was required to reach a statistically significant reduction of the HTT1a smear in the siRNA MSH3-1000 treated groups (Fig. 4D, *P* = 0.0395, *t* = 2.289, df = 13, two-tailed unpaired *t*-test, *N* = 8 NTC and 7 MSH3). When data from all groups were combined, there was a significant positive correlation between the intensity of the HTT1a smear and the reduction in MSH3 protein levels (Fig. 4E, *R*^2^ = 0.3033, *P* = 0.0003, *n* = 19–20 mice per group). In the same mice, there was no change in levels of full-length HTT. These results suggest that production of the HTT1a smear detected by 1B12 is affected by levels of MSH3, which is known to modulate CAG repeat expansion. Uncropped blots for mouse data are shown in Supplementary Figs 5–9.

### 1B12 and 11G2 do not detect immunoreactive HTT1a by western blot in lysates from Huntington’s disease brain and human HD109Q neurons

*HTT1a* mRNA has been detected in some samples from human Huntington’s disease post-mortem brain and patient fibroblasts.^9^ Therefore, we applied similar methods of analysis used in Huntington’s disease mice to examine Huntington’s disease post-mortem brain. In S1 and CH fractions that were separated in 3–8% Tris-acetate (Supplementary Fig. 4A and B) or 12% Bis-Tris (Supplementary Fig. 4C) gels, 1B12 antibody detected a doublet at 56–60 kDa that was more prevalent in the human Huntington’s disease putamen and cortex than in control brain. These bands were not blocked when 1B12 antibody was preincubated with the C-terminal HTT1a blocking peptide (Supplementary Fig. 4A). The 56–60 kDa bands also appeared in post-mortem cortex from three Parkinson’s disease patients (Supplementary Fig. 4C) suggesting that the non-specific band may be elevated in disease brain. Immunoprecipitation with 1B12 or 11G2 from human Huntington’s disease brain lysates of adult-onset HD patients with 42 and 53 CAG repeats in the *HTT* allele and re-probing with 2B7 antibody failed to detect HTT immunoreactive proteins. However, using similar assay conditions, proteins immunoprecipitated with 2B7 antibody expressed full-length WT and mutant HTT when detected with Ab1 antibody (Supplementary Fig. 4D–F). Finally, we examined lysates from iPSC-derived medium spiny neurons and cortical neurons in WA09 and HD109Q lines. Despite the higher CAG repeat in the HD109Q line, only the 56 kDa band was detected in both control WA09 and HD109Q lines (Supplementary Fig. 4G). Altogether these findings suggested that unlike Huntington’s disease mouse brain, 1B12 and 11G2 antibodies did not detect HTT1a in human Huntington’s disease brain or in iPSC-derived human HD109Q neurons by western blot assays. Uncropped blots for human data are shown in Supplementary Fig. 10.

## Discussion

Aberrant splicing between exon 1 and exon 2 of *HTT* due to the presence of a cryptic polyadenylation in intron 1 produces an exon 1 mRNA named *HTT1a* and encodes protein HTT1a that terminates at a proline residue (aa 90, based on 23 CAGs).^1,9^ This fragment was identified as translating the endogenously generated HTT exon 1 protein product that may nucleate and recruit aggregates. *HTT1a* mRNA has been detected in human Huntington’s disease brain, in a wide range of human peripheral tissues and in Huntington’s disease knock-in mouse models that express human exon 1 and its levels of expression are CAG repeat length dependent.^9,14,39^ HTT1a protein levels in human and mouse Huntington’s disease brain have been assessed using immunoprecipitation, HTRF, MSD and AlphaLISA assays with paired N-terminal HTT antibodies that individually are not specific for the endogenous protein by western blot.^3,9,40,41^ Here, we showed that direct western blot assays can be used to detect HTT1a in lysates from Huntington’s disease transgenic (R6/2, YAC128) and Huntington’s disease knock-in mouse brain using neoepitope-specific monoclonal antibodies P90-1B12 and 11G2 which were directed to the C-terminal eight amino acids of HTT exon 1 protein.^16,17^ Six-month-old mice from the allelic series of Huntington’s disease knock-in mouse models resolved HTT1a as discrete bands and as prominent HMM smears that varied in migration and signal intensity in relation to CAG repeat length and age of the mice. The smears appeared in 6-month-old Q111, Q140 and Q175 mice, consistent with the presence of aggregates that are prevalent by this age with immunostaining^13,42-44^ and in 4- and 8-month-old YAC128 mouse striatum concurrent with loss of striatal volume at 3 months of age^45^ and appearance of EM48-positive nuclear aggregates by immunostaining at 2 months.^46^ Significant lowering of MSH3, a CAG repeat modifier, in caudate putamen of 5-month-old Q111 mice reduced the HTT1a smear suggesting that the intensity of the smear depends on CAG repeat expansion. Altogether, the present results support the presence of HTT1a in mouse Huntington’s disease brain and the feasibility of using western blot detection of HTT1a in Huntington’s disease mice as a biomarker to test the effects of agents that limit CAG repeat expansion.

The HTT1a smear seen in Huntington’s disease mice with 1B12 and 11G2 antibodies may partly correspond to SDS insoluble products identified in Q175 and R6/2 mice using other biochemical assays. HMM products migrating above 300 kDa were found in nuclear fractions from R6/2 mouse brain by agarose gel electrophoresis for resolving aggregates (AGERA) and probed with antibodies 4C9 and MW8.^12^ Also, in nuclear fractions from brain lysates of R6/2 mice SDS insoluble products remained at the top of a western blot probed with S830 antibody.^12^ Consistent with these protein studies, a screen of antibodies by Bayram-Weston *et al*.^47^ showed that MW8 and S830 antibodies were the most sensitive for identifying nuclear inclusions and diffuse staining in Huntington’s disease transgenic and knock-in mice. The decline in HTT1a smear observed by western blot after lowering *MSH3* mRNA contrasts with the limited effects on aggregate formation seen by immunostaining after silencing *MSH3* mRNA in Q111 mice and Q175 mice.^38,48^ This suggests the HTT1a smear detected by 1B12 and 11G2 in western blot is a fraction of the aggregated mutant HTT present in tissue.

Factors that could explain why 1B12 and 11G2 antibodies did not detect HTT1a in the human Huntington’s disease brain are that protein levels are too low due to marked neuronal loss and/or the protein is sequestered into a nuclear compartment that is not accessible. Also, based on our findings in Huntington’s disease knock-in mice, the CAG repeat size may need to reach 80 to detect the HTT1a band and at least 100 to see a smear. Moreover, immunoprecipitation of HTT1a with the 1B12 and 11G2 antibodies from lysates of human as well as mouse Huntington’s disease brain was unsuccessful using assay conditions that pull down and enrich for full-length HTT with an anti-HTT N-terminal antibody. The marked loss of neurons in Huntington’s disease patient neostriatum and cortex compared to Huntington’s disease knock-in mouse and the proliferation of glial cells which are reported to have less somatic instability than neurons^49,50^ could limit levels of HTT1a in human Huntington’s disease brain. Also, RNA sequencing analysis of human Huntington’s disease neostriatum suggests that very large somatic expansions that may be required to detect HTT1a are infrequent and short-lived.^51^ In studies of cortex using single serial fluorescence-activated nuclear sorting (sFANS) and single-nucleus RNA sequencing, layer 5a pyramidal neurons in Huntington’s disease post-mortem cortex exhibit CAG repeat expansions.^51,52^ Since these neurons were reported as lost early in disease, they may be sparse in our post-mortem samples from the cortex.

In summary, our results showed that monoclonal 1B12 and 11G2 antibodies are useful for direct western blot detection of HTT1a in subcellular fractions from brains of Huntington’s disease knock-in mice. The sizes and solubility of HTT1a varied with subcellular compartment, age and CAG repeat expansion, and these features could be easily compared. Relatively small amounts of tissue are required for detection of HTT1a by SDS-PAGE and western blot, allowing tissues for other assays to be collected from the same mouse brain. Moreover, the ability to quantify changes in levels of HTT1a smear in western blots by reducing levels of MSH3 will be a useful readout for preclinical studies.

## Supplementary Material

fcaf314_Supplementary_Data

## Data Availability

The authors confirm that the data supporting the findings of this study are available within the article and its supplementary material. Raw data are available upon request from the corresponding author.
